# Rescripting Suicidal Mental Imagery in Young People: A Pilot Study of Feasibility, Acceptability and Safety

**DOI:** 10.3390/bs16101954

**Published:** 2026-10-20

**Authors:** Lianne Schmaal, Georgie Paulik, Zamantha Munoz, Laura S. van Velzen, Pemma Davies, Martin Mitrevski, Eleanor Bailey, Phoebe Gleeson, Lisa Incerti, Samantha Galea, Benjamin Dorevitch, Jaymee Pingitore, Sarah Bendall, Connie Markulev

**Affiliations:** 1 Centre for Youth Mental Health, University of Melbourne, Melbourne, VIC 3010, Australia; zamantha.munoz@orygen.org.au (Z.M.); laurasvanvelzen@gmail.com (L.S.v.V.); eleanor.bailey@orygen.org.au (E.B.); sarah.bendall@orygen.org.au (S.B.); connie.markulev@orygen.org.au (C.M.); 2 Orygen, The National Centre of Excellence in Youth Mental Health, Parkville, VIC 3052, Australia; pemma.davies@gmail.com; 3 Perth Voices Clinic, Murdoch, WA 6150, Australia; georgiepaulik@perthvoicesclinic.com.au; 4 School of Psychology, Murdoch University, Murdoch, WA 6150, Australia; 5 School of Psychology, Curtin University, Curtin, WA 6102, Australia; 6 Orygen Specialist Program, Melbourne Health (now PYMHWS), Melbourne, VIC 3052, Australia; martin.m.mitrevski@gmail.com (M.M.); phoebe.gleeson@pymhws.org.au (P.G.); lisa.incerti@pymhws.org.au (L.I.); samantha.galea@pymhws.org.au (S.G.); benji.dorevitch@pymhws.org.au (B.D.); jaymee.pingitore@pymhws.org.au (J.P.); 7 Parkville Youth Mental Health and Wellbeing Service (PYMHWS), Parkville, VIC 3052, Australia

**Keywords:** suicidal ideation, mental imagery, imagery rescripting, intrusive cognitions, mood disorders, adolescence, young adulthood

## Abstract

Suicidal mental imagery—vivid, intrusive mental images of suicide or death—is common among young people experiencing suicidal ideation and may increase risk of suicidal behaviour, yet interventions directly targeting these images are limited. We conducted a single-arm pilot study evaluating the feasibility, acceptability and safety of a brief, therapist-delivered imagery rescripting intervention targeting action-facilitating suicidal mental imagery in help-seeking young people aged 16–25 years with mood disorders and current or recent suicidal ideation. Fifteen participants were recruited; 12 were eligible for the intervention and 11 (92%) completed treatment and follow-up. The recruitment target (*N* = 25) was not met, largely due to service disruptions and clinician capacity constraints. Acceptability and safety criteria were met: 73% reported that sessions were not distressing, none felt less safe following treatment, and no participant showed increased suicidal ideation from baseline to follow-up. Two serious adverse events occurred but were judged unrelated to the intervention. Exploratory analyses showed reductions in suicidal-image frequency, realness, compellingness, intrusiveness and distress, alongside a 62.5% reduction in suicidal ideation severity. Our pilot study provides preliminary indications that directly rescripting action-facilitating suicidal mental imagery showed encouraging short-term safety and acceptability signals in young people, supporting evaluation in a larger randomised controlled trial.

## 1. Introduction

Suicide is the third leading cause of death worldwide among individuals aged 15–29 (World Health Organisation, 2025) and the leading cause of death among people aged 15–24 years in Australia (Australian Institute of Health and Welfare, 2026). Suicide has devastating ripple effects on families and communities, highlighting the need to improve the identification, prevention, and treatment of suicidal thoughts and behaviours in young people. Despite substantial investment and research into youth suicide prevention, high-quality targeted interventions that reduce suicidal behaviour in youth remain sparse (Witt et al., 2021). For example, a recent systematic review concluded that multi-component treatment such as dialectical behaviour therapy (DBT) shows moderate evidence for reducing suicidal ideation but insufficient evidence of effectiveness for reducing suicidal behaviours, while other psychosocial treatments showed insufficient to low strength of evidence for reducing suicidal outcomes (Sim et al., 2025). This is consistent with earlier reviews highlighting the need for further development and evaluation of interventions that target specific mechanisms underlying suicidal thoughts and behaviours, rather than relying solely on treatments directed at broader psychiatric symptoms (Busby et al., 2020; Riblet et al., 2017; Witt et al., 2021). Therefore, the quest to identify a novel, focused intervention target that could have wide positive impacts on suicidal thoughts and behaviours has been a priority in youth suicide research.

One potentially important but under-recognised component of suicidal cognition that could be specifically targeted using focused treatment is mental imagery. Suicide risk assessment and psychological interventions have traditionally focused on verbal thoughts about death, suicide and self-harm (Andreotti et al., 2020). However, suicidal cognition can also represent mental (visual) imagery: perceptual-like experiences occurring in the absence of corresponding external sensory input (e.g., seeing a picture in the mind’s eye, despite not receiving this information from the immediate setting; Ji et al., 2019). Mental imagery may have a particularly strong relationship with emotion, motivation and behaviour (Holmes & Mathews, 2010) compared with verbal–linguistic cognition. Consequently, dysfunctional mental imagery has been suggested to be integral to the maintenance of many psychological disorders (Çili & Stopa, 2015; Shapiro, 2018). The distinction between verbal thought and imagery may be particularly relevant to suicidal cognition, where vivid mental representations of suicide or death may convey experiential and emotional information that is not captured by asking about suicidal thoughts alone.

Mental images of suicide are described as realistic, compelling, vivid and detailed images of being dead, or of plans for future suicide attempts (Holmes et al., 2007b). Early research described these experiences as “flash-forwards” because of their similarities to the vivid, intrusive flashbacks observed in PTSD, but with the imagery directed towards an anticipated future event (Holmes et al., 2007b). Subsequent research has demonstrated that suicidal mental imagery is very common among adults with mood disorders and suicidal ideation, with prevalence estimates ranging from ~75–90% in clinical samples (Lawrence et al., 2023). The very few studies examining the prevalence and characteristics of suicidal mental images in young people report prevalence rates between 63 and 98% in clinical samples of adolescents or young adults with suicidal cognitions and/or self-harm behaviour (De Rozario et al., 2021; Lawrence et al., 2021, 2023; Schmied et al., 2024; Susi et al., 2024). In our previous study of 33 help-seeking young people with suicidal cognitions, suicidal mental images were described as real (85%), vivid (91%), intrusive (61%) and distressing (79%) (De Rozario et al., 2021).

Importantly, suicidal mental images may increase risk of transitioning from ideation to attempt, as construction of mental images of future events has been shown to make these events more probable and to promote action (Gregory et al., 1982; Libby et al., 2007; Szpunar et al., 2013). Emerging evidence shows that suicidal and self-harm mental imagery can differentiate between those who only think about suicide and those who attempt suicide, over and above suicidal verbal thoughts (Bayliss et al., 2025; Cleare & O’Connor, 2026; De Rozario et al., 2021; Lawrence et al., 2021, 2023; Wetherall et al., 2018). Suicidal mental imagery has also been found to be predictive of future suicidal ideation and urges to self-harm, as well as increased likelihood of acting on these urges (Bayliss et al., 2024; Ji et al., 2024). Several pathways may link mental imagery to this progression from suicidal ideation to behaviour. Suicidal mental imagery allows individuals to imagine possible future events and their consequences, potentially influencing motivation and subsequent behaviour (Schacter et al., 2012). For some individuals, particularly when imagery is compelling and action-facilitating (i.e., motivates the person to act on the image), repeated suicidal imagery may function as a form of cognitive rehearsal for suicidal behaviour. This is consistent with the integrated motivational–volitional model, which distinguishes factors that contribute to the emergence of suicidal ideation from ‘volitional’ factors that increase the likelihood that suicidal thoughts are translated into suicidal behaviour; suicidal mental imagery is proposed to be one such volitional factor by increasing the cognitive accessibility and perceived enactability of suicide (Cleare & O’Connor, 2026; O’Connor & Kirtley, 2018). In addition, the accumulated, repeated experience of visually imagining one’s own future suicide attempt or death could lower fear of death and increase pain tolerance, thereby increasing capability for suicide (Bayliss et al., 2024; Joiner et al., 2005; O’Connor & Nock, 2014).

Taken together, the high prevalence, vividness and intrusiveness of suicidal mental imagery, and its apparent role in lowering the threshold for suicidal behaviour, make it a compelling and novel treatment target. Imagery-based psychological interventions aim to directly target distressing mental images and include Imaginal Exposure, Functional Imagery Training, Positive Imagery, Eye Movement Desensitisation and Reprocessing Therapy (EMDR), and Imagery Rescripting (Holmes et al., 2007a). A randomised controlled clinical trial showed reductions in the frequency and severity of suicidal mental images and suicidal ideation following a brief cognitive dual-task intervention involving eye movements in adults with depressive symptoms and distressing intrusive suicidal mental images (Van Bentum et al., 2024). This intervention, which largely followed an EMDR procedure, directly targeted the suicidal image with the aim of reducing its emotional intensity and intrusiveness. In addition, in a randomised controlled feasibility trial in university students, a guided positive mental imagery intervention was found to be feasible, safe and acceptable and showed preliminary effectiveness (Palmier-Claus et al., 2025). However, neither approach deliberately transforms the content, meaning or outcome of the suicidal mental image itself.

We have previously proposed imagery rescripting (ImRs) as a novel and promising intervention to directly transform the content of the suicidal imagery (Paulik et al., 2024). ImRs is a psychological intervention aimed at treating aversive or dysfunctional mental images or memories, and has been implemented as a stand-alone treatment or within various treatment modalities, such as cognitive behavioural therapy and schema therapy (Arntz, 2012; Wild et al., 2007). During ImRs, an individual activates a distressing mental image or memory and, with a clinician, develops and vividly rehearses an alternative sequence or outcome which meets their underlying emotional needs. The intervention therefore works directly with the content and emotional meaning of an image rather than focusing exclusively on its associated verbal propositions (Arntz, 2012; Arntz & Weertman, 1999; Holmes et al., 2019). ImRs has been applied to distressing mental imagery across a range of psychological disorders, and a meta-analysis of 23 trials involving 805 adults found that ImRs was associated with reductions in symptoms linked to aversive mental imagery (Kroener et al., 2023). ImRs may be particularly suited to suicidal imagery because it explicitly targets the personally meaningful content and anticipated outcome of the image, identifying the underlying unmet emotional needs and deliberately rehearsing alternative, adaptive responses that address those needs. A recent single case series study showed initial evidence that ImRs of self-harm mental images is feasible and potentially beneficial for adolescents engaging in self-harm (Schmied et al., 2024). However, ImRs has not yet been tested or applied to mental images of future suicide or death.

In this pilot study, we aimed to determine the feasibility, acceptability and safety of a brief, therapist-delivered ImRs intervention targeting action-facilitating suicidal mental imagery in help-seeking young people aged 16–25 years experiencing current or recent suicidal ideation with suicidal mental imagery. We adapted existing frameworks for ImRs targeting other types of intrusive imagery associated with psychological disorders, including major depressive disorders, anxiety disorders, obsessive-compulsive disorders, and trauma-affected voice-hearing (see Arntz, 2012; Morina et al., 2017 for reviews). Given the heterogeneous functions of suicidal imagery, it is important to establish whether young people can safely and acceptably engage with an intervention that directly activates and modifies an action-facilitating suicide-related mental image.

## 2. Methods

### 2.1. Design

This study was a single-arm pilot study of a novel ImRs intervention targeting action-facilitating suicidal mental images in help-seeking young people with mood disorders and current suicidal ideation who experience suicidal mental imagery. The pilot study was approved by the Royal Melbourne Hospital Human Research Ethics Committee (HREC Ref No. 82363/2022.042) on 30 November 2022. The study was registered on the Australian New Zealand Clinical Trials Registry (ANZCTR, identifier ACTRN12624000639527) on 17 May 2024. Recruitment commenced with the first participant’s first visit (FPFV) on 5 June 2023; registration therefore occurred retrospectively (i.e., after FPFV) but prior to data analysis. The feasibility, acceptability and safety thresholds reported in this manuscript were prespecified in the study protocol dated 24 November 2022 (approved by the ethics committee) and were recorded prior to data analysis and FPFV.

### 2.2. Participants

Fifteen help-seeking young people attending a specialised youth mental health service for complex and severe mood disorders in Melbourne (Rice et al., 2022) participated in this study. Inclusion criteria were: (1) Aged between 16 and 25 years (inclusive); (2) Having current or recent suicidal ideation (within the last 4 weeks determined by the Columbia Suicide Severity Rating Scale (CSSRS; Posner et al., 2011); (3) Engagement with treatment as determined by their treating clinician; (4) Familiarity with, and willingness to use available crisis supports in context of suicide risk determined by patient report and clinical information; (5) Ability to provide informed consent and comply with study procedures; (6) Willingness to nominate an emergency contact; (7) Reporting one or more mental images of suicide, which the young person indicates increases risk for suicidal behaviour (action-facilitating) given that images that inhibit suicidal actions may be protective, in the past 14 days; (8) Willingness to dedicate two treatment sessions to ImRs therapy to treat the suicidal mental image (with an optional third and fourth session). Exclusion criteria were: (1) Presence of an intellectual disability as documented on their medical record; (2) Not able to converse in, or read in, English; (3) Limited engagement in face-to-face treatment or approaching discharge as determined by the treating clinician; (4) Currently at acute risk of suicide requiring acute risk management, as determined by the treating clinician; (5) Actively manic or psychotic as assessed by the treating clinician; (6) Presence of organic brain disease or severe neurological impairment, as documented in medical record; (7) Experience of only mental image(s) of suicide which decreases the risk of suicidal behaviour (i.e., a protective image) as reported by the participant.

### 2.3. Procedure

Recruitment took place between January 2023 and November 2025. Potentially eligible participants were screened and referred to the study by their treating clinicians who were trained in delivering the intervention. Initial screening included the treating clinician and young person discussing whether the image had occurred in the last 14 days and whether the participant perceived it as increasing their motivation to engage in suicidal behaviour (action-facilitating). They were then contacted by a research team member and sent the study documentation, including a detailed description of the procedures, aims, risks, and benefits. The research assistant reviewed participants’ medical records to determine broad inclusion/exclusion criteria and contacted the young person to schedule a screening and informed consent meeting. During the informed consent, the research assistant presented the aims and procedures of the study (verbally and in written text), and the process for making enquiries, complaints and withdrawals. Of 25 potentially eligible young people referred by the clinicians, fifteen were broadly screened and provided informed written consent. For participants aged 16 or 17, additional consent from the nominated parent or legal guardian was obtained. Participants engaged in a baseline assessment (T1), 2–4 imagery rescripting sessions (T2–T5; intervention), and a follow-up assessment within one week of completion of the intervention (T6).

Following consent and during baseline screening, there was a structured assessment using the Suicidal Flashforwards and Cognitions Interview (SCFI) (Holmes et al., 2007a, 2007b). The SCFI items were used to probe whether the image (a) involved a future suicidal act, (b) was experienced as compelling or rehearsal, and (c) whether the participant reported the image increases the likelihood of acting or serves as a “how to” or rehearsal. The following decision rule was applied: an image was classified as action-facilitating if the young person endorsed that the image (i) increased their urge to act or (ii) made a suicidal act seem more likely or possible, or (iii) provided behavioural detail of the rehearsal that the participant felt could facilitate action. Images described as comforting, life-affirming, or explicitly discouraging suicidal action were classified as protective and excluded. Clinicians were trained on these criteria and, where there was uncertainty, the clinician discussed the case with the study team to reach consensus.

The intervention was delivered within participants’ existing clinical care, with usual treating-team risk-management procedures maintained throughout the study. Acute suicide risk was assessed by the clinician at the beginning of each intervention session, and sessions were postponed if acute risk was identified. Clinicians could also pause or discontinue imagery work at any time if risk, distress, engagement, or other clinical circumstances indicated that continuation was inappropriate. Distress was assessed at the start and again at the end of each ImR session using the Subjective Units of Distress Scale (SUDS; Wolpe, 1969; 0 = no distress to 10 = extreme distress). Where distress was present, breathing exercises and safe-place imagery were used to support emotional regulation.

### 2.4. Intervention

The ImRs intervention (sessions T2–T5) was delivered by the participant’s treating clinician at the specialised youth mental health service. Prior to study commencement, six clinicians were trained in ImRs by a senior clinician with expertise in ImRs (author GP). Study clinicians were all experienced clinical psychologists. Training was delivered over two days of face-to-face training, incorporating didactic teaching, video demonstration and role-play, and was supplemented by a webinar and ongoing clinical supervision. The first intervention session (T2) with the clinician included psychoeducation around mental imagery in general and mental images of suicide specifically, an explanation of the intervention, and development of safe place imagery, which could be used during the rescripting sessions to lower distress if necessary. The treating clinician also completed the Suicidal Flashforwards and Cognitions Interview (SCFI; Holmes et al., 2007b) with the participant, which assesses mental imagery of suicide as well as verbal suicidal thoughts. The SCFI is a structured interview developed to assess suicidal mental imagery and associated cognitions; it has been used in prior imagery research, although large-scale psychometric data remain limited (De Rozario et al., 2021; Holmes et al., 2007a). During the SCFI, participants rate how much time they spent preoccupied with suicide-related imagery/verbal thoughts, and how compelling, real, vivid, intrusive and distressing their suicide-related imagery/verbal thoughts are, from which perspective they view the mental images and whether they also experience positive mental images.

The clinician and participant together selected a suicidal mental image the participant experienced in the last 14 days to be targeted by ImRs. It was important that the suicidal mental image selected for rescripting was one that motivated the participant towards suicidal behaviour (facilitating), rather than one that inhibited suicidal action (protective). This required a case-by-case assessment of whether the image functioned as a facilitating, intrusive flash-forward or escape-based fantasy, and whether it motivated the individual towards or away from suicidal behaviour (Paulik et al., 2024). The clinician also explored the context in which the image occurred, its emotional and interpersonal meaning, and the needs or concerns represented within the image to develop an individualised formulation of its function. The image was described in detail by the participant as part of the SCFI. This mental image was then rescripted in the following one or two sessions (T3 and T4), with an optional fourth session (T5) if more time was needed to practice the rescripting or if there was a second mental image of suicide that the participant wanted to rescript. During rescripting, the participant was supported to activate and describe the target image, then collaboratively develop and vividly rehearse an alternative sequence or outcome. The aim was not simply to replace the suicidal image with a positive image, but to develop an alternative that acknowledged the participant’s emotional experience and addressed the meaning or unmet needs associated with the original image, while introducing possibilities for safety, coping, connection or other adaptive responses.

### 2.5. Measures

Baseline assessment (T1) included the following measures:

- Demographics including sex, gender, age, living arrangements, nationality, education and employment status;
- Diagnostic status was assessed at baseline using the Mini International Neuropsychiatric Interview (MINI), which is a brief structured diagnostic interview developed to assess psychiatric disorders according to DSM and ICD diagnostic criteria (Sheehan et al., 1998). The MINI was administered by a research assistant trained in MINI administration, to confirm a current diagnosis of major depressive disorder and to determine the presence of past or current (hypo)manic episodes. A current manic or psychotic episode, as judged by the treating clinician, not the MINI, was an exclusion criterion (Section 2.2); a lifetime history of (hypo)manic symptoms was not.
- The Columbia-Suicide Severity Rating Scale (C-SSRS; Posner et al., 2011) assesses the intensity (ranging from wish to be dead, non-specific active thoughts, thoughts with method but no plan, some intent without a specific plan, and plan and intent) and frequency of suicidal ideation and information regarding past suicide attempts and self-harm. The CSSRS is a widely used structured interview with demonstrated inter-rater reliability and convergent and predictive validity. Psychometric evidence has been demonstrated in adolescent samples, including evidence that severity and intensity of suicidal ideation predict subsequent suicidal behaviour (Gipson et al., 2015; Posner et al., 2011).
- The Quick Inventory of Depressive Symptomatology (QIDS; Rush et al., 2003) is a 16-item self-report questionnaire measuring symptom severity of depression. The QIDS-SR16 has demonstrated good internal consistency and construct validity in adolescent samples and has shown good correspondence with clinician-rated measures of depressive symptoms, supporting its use in adolescent clinical populations (Bernstein et al., 2010; Zhang et al., 2020).
- The Altman Self-Rating Mania Scale (ASRM; Altman et al., 1997) is a 5-item self-report measure assessing the presence and severity of hypomanic or manic symptoms. The ASRM has demonstrated good reliability and concurrent validity in its original validation study (Altman et al., 1997) and is included by the American Psychiatric Association as the DSM-5-TR Level 2—Mania measure for children and adolescents aged 11–17 years, supporting its use for assessing manic symptoms in this age group (American Psychiatric Association, 2022).
- The Overall Anxiety Severity and Impairment Scale (OASIS; Norman et al., 2006) is a 5-item self-report questionnaire assessing anxiety symptom severity and associated impairment. The OASIS has demonstrated good internal consistency, test–retest reliability and convergent validity in adult clinical and non-clinical samples, and recent psychometric evaluation in 598 adolescents demonstrated a unidimensional structure, excellent internal consistency and construct validity, supporting its use in adolescent populations (Maçãs-Carvalho et al., 2025; Norman et al., 2006).
- Spontaneous Use of Imagery Scale (SUIS; Nelis et al., 2014) is a 12-item self-report questionnaire measuring the frequency and likelihood of spontaneous visual mental imagery in daily life, with higher scores reflecting a greater trait propensity for mental imagery. Psychometric evaluation supports acceptable internal consistency and construct validity (Nelis et al., 2014). An adapted seven-item version has also demonstrated acceptable internal consistency in adolescent samples, providing preliminary evidence for the measure’s applicability to young people (de Voogd et al., 2017).

Outcome measures (T6) included:

- A short debriefing questionnaire to determine the acceptability of the intervention to participants, including 16 items covering the level of distress experienced by the research assessment and ImRs sessions, whether speaking about or rescripting the suicidal mental images impacted on suicidal feelings, and whether participants experienced participating in the study and intervention as meaningful. Scores ranged from 1 (“not at all”) to 5 (“very much”), with a score of 3 being “neutral”. For the acceptability and safety criteria, a score of 4 or 5 was considered an increase in distress and unsafety, respectively.
- The SCFI, C-SSRS, OASIS and QIDS were re-administered at follow-up to assess effects of the intervention on suicidal imagery characteristics, overall severity of suicidal ideation and severity of depressive and anxiety symptoms in exploratory analyses.

Data on concurrent treatments (including medication changes, treatment intensity, crisis contacts) were not systematically collected. Details regarding any hospital admissions were collected via clinician routine reporting of adverse events.

### 2.6. Youth Participation and Consultation

The treatment protocol, participant information and consent form were adapted based on two rounds of feedback from two young people with lived experience of suicidal ideation and suicidal mental imagery.

### 2.7. Analyses

Descriptive statistics were used to characterise the sample. Differences between frequency, vividness, intrusiveness, realness and distressing effects of suicidal mental images versus verbal thoughts of suicide were assessed using paired sample *t*-tests. Acceptability, feasibility and safety markers were presented as numbers and percentages including Wilson’s 95% confidence intervals, and compared to prespecified criteria (Table 1). Exploratory paired sample *t*-tests examined changes in frequency, vividness, intrusiveness, realness and distressing effects of suicidal mental images, as well as changes in intensity of suicidal ideation and severity of depressive and anxiety symptoms. For the eight continuous pre-post outcomes (QIDS-SR-16, OASIS, and the six SCFI image characteristics), change was assessed with paired-samples *t*-tests. Percentage change was calculated as the mean within-person difference (follow-up minus baseline) expressed as a percentage of the baseline mean. 95% confidence intervals for this percentage were obtained by rescaling the confidence interval of the raw mean difference (derived from the paired *t*-test, using the t-distribution with *n* − 1 degrees of freedom) by the same baseline-mean denominator. Effect sizes were calculated as Cohen’s dz, the standardised mean paired difference (mean difference score divided by the standard deviation of the difference scores), the variant recommended for within-subject designs.

Because the C-SSRS suicidal ideation severity item was measured on an ordinal 6-point scale, its change was instead assessed with a Wilcoxon signed-rank test. Percentage change for this outcome was calculated from the Hodges–Lehmann estimate of the median paired difference, with its 95% confidence interval taken directly from the Wilcoxon test. The effect size is the matched-pairs rank-biserial correlation (r), computed from the sum of positive and negative signed ranks. Given the small sample size (n = 11), all pre-post comparisons should be interpreted as preliminary estimates rather than confirmatory tests.

To contextualise the safety outcomes observed in this study, rates of suicide attempts and new-onset non-suicidal self-harm were compared against those recorded in a file audit of clients from the same youth mental health service from which participants were recruited (O’Gorman et al., 2023; Rice et al., 2022). Because file audit events are recorded over each client’s full episode of care, which is not directly comparable to this study’s shorter baseline-to-follow-up window, we constructed a duration-matched comparison sample rather than comparing against the full audit cohort. For each participant, the number of days between baseline and follow-up assessment was calculated. File audit records were then restricted to clients with an episode-of-care duration within the observed range of these intervals (20–139 days), and nearest-neighbour matching (Mahalanobis distance, three audit records matched per participant, with replacement) was used to select file audit comparators with a similar duration of care for each participant. Suicide-attempt and new-onset non-suicidal self-harm rates were then calculated within this duration-matched subsample. This matching procedure yielded a comparison subsample of 19 file audit records. The duration-matching procedure itself was not prespecified but introduced because clients in the file audit typically have longer episodes of care than this study’s follow-up window, giving more opportunity to engage in self-harm or attempt suicide, and restricting the file audit to a comparable duration of care provided a fairer basis for comparison.

## 3. Results

### 3.1. Participants

**Figure 1:**
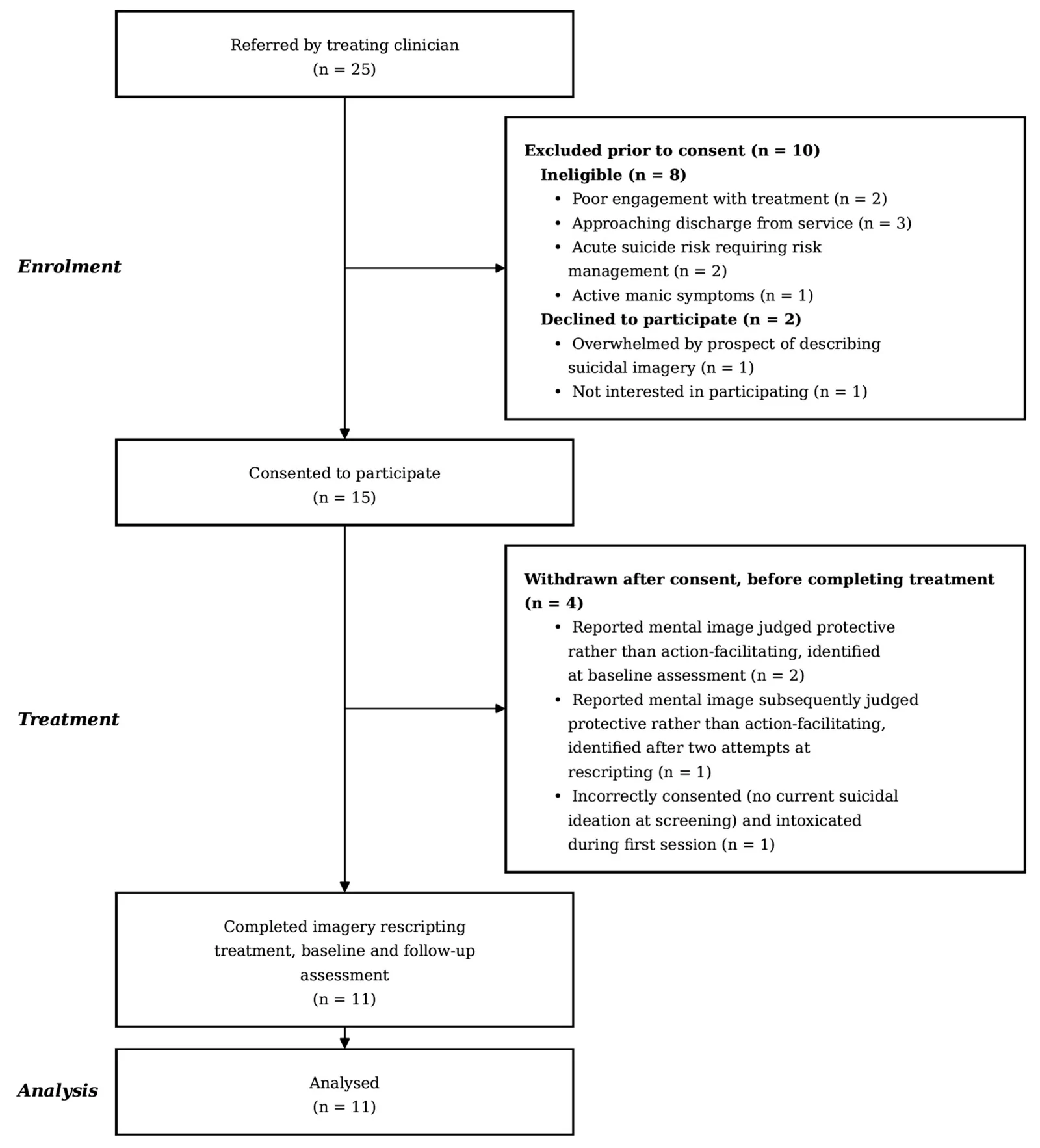
Consort diagram.

Two of the 15 participants indicated during the baseline assessment that their suicidal mental image was protective rather than action-facilitating; thus, they were deemed ineligible for the imagery rescripting intervention. One participant discontinued the intervention after two attempts at rescripting. Although the target image was initially identified as action-facilitating, the participant subsequently described the image as serving an adaptive or personally meaningful function and did not experience proposed alternative scripts as credible or therapeutically helpful. Following collaborative discussion, the participant and clinician agreed that further rescripting was unlikely to be beneficial. One participant was excluded because they had been incorrectly consented despite not meeting the inclusion criterion of current suicidal ideation at screening and were also found to be intoxicated during the intervention (Figure 1).

Thus, 11 participants received the full intervention: seven completed T2 (psychoeducation around suicidal mental imagery, explanation of the intervention, and development of safe place imagery) plus one imagery rescripting session (T3), and four completed T2 plus two imagery rescripting sessions (T3 and T4). Sample characteristics can be found in Table 2.

### 3.2. Suicidal Mental Imagery

**Figure 2:**
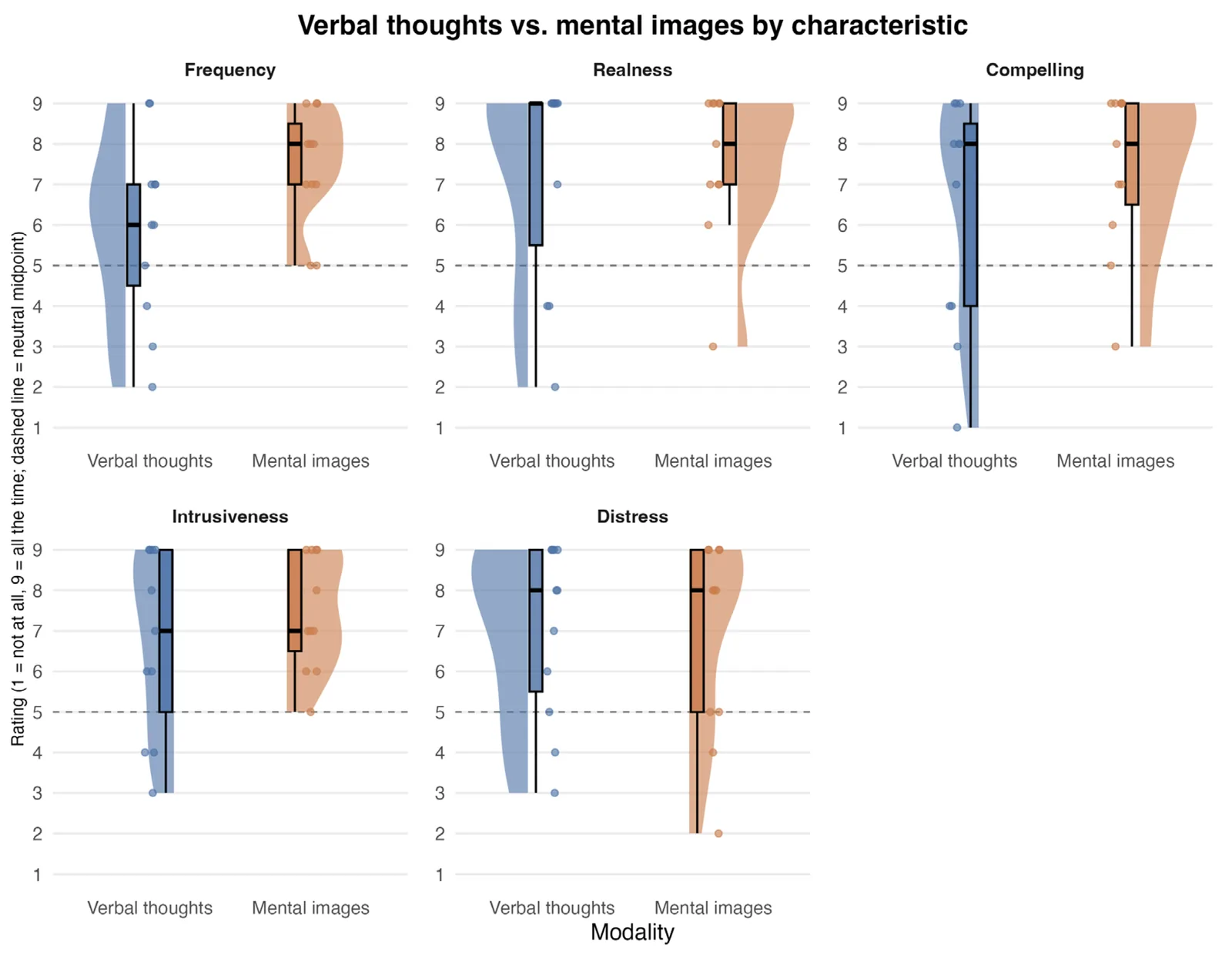
Comparison of verbal suicidal thoughts and suicidal mental images on ratings of their frequency, realness, compellingness, intrusiveness, and distress.

Suicidal mental images were experienced very frequently, as real, vivid, intrusive and distressing (Figure 2). Compared to their verbal thoughts of suicide, participants rated their mental images as significantly more frequent (*t*(10) = 2.42, *p* = 0.036, *d* = 0.73), but not significantly more real (*t*(10) = 0.49, *p* = 0.635, *d* = 0.15), compelling (*t*(10) = 1.29, *p* = 0.226, *d* = 0.39), intrusive (*t*(10) = 1.44, *p* = 0.181, *d* = 0.43), or distressing (*t*(10) = 0.16, *p* = 0.875, *d* = 0.05) (Figure 2). All participants reported that their suicidal mental images were images of a future suicide attempt, experienced from a first-person perspective by 4 participants, a third-person perspective by 4 participants, and a mix of both by 3 participants. Five participants also indicated having positive images of their future self, while 6 participants never experienced positive future-self imagery.

### 3.3. Primary Outcomes

#### 3.3.1. Feasibility

Of the target sample of 25 participants, 15 were recruited and consented into the study (60% of target). The pre-specified feasibility criterion of reaching N = 25 was therefore not met. Recruitment fell short of target for several reasons. First, although six clinicians were trained to deliver the imagery rescripting intervention, staff turnover, changes in clinical roles and periods of extended leave reduced clinician availability over the recruitment period. Second, eligibility required participants to have experienced recent action-facilitating suicidal imagery, while excluding those whose current suicide risk required more acute clinical management. This created a relatively narrow window for recruitment and reduced the number of otherwise potentially suitable clients who were eligible at screening. Third, because participating clinicians were concurrently acting as participants’ case managers, clinicians’ competing acute-care and case-management responsibilities frequently limited capacity to schedule study intervention sessions.

Of the 15 participants consented into the study, during the baseline SCFI, 2 participants were deemed ineligible for the imagery rescripting intervention because their suicidal mental image was protective rather than action-facilitating. One further participant was withdrawn: they had been incorrectly consented despite not meeting the inclusion criterion of current suicidal ideation at screening and were also found to be intoxicated during the intervention. This resulted in 12 participants eligible for the intervention. Of the 12 participants eligible for the intervention, 11 completed both the introductory intervention session (T2) and at least one imagery rescripting session (T3–T5), as well as the baseline and follow-up assessments. This equates to 11/15 = 73.3% completion among all consented participants and 11/12 = 91.7% completion among participants eligible for the ImRs intervention. Because the feasibility criterion in Table 1 was prespecified using the consented denominator (>80% of consented participants), the prespecified threshold was not met (73.3% < 80%), although completion among those eligible for the intervention exceeded the threshold.

#### 3.3.2. Acceptability

**Figure 3:**
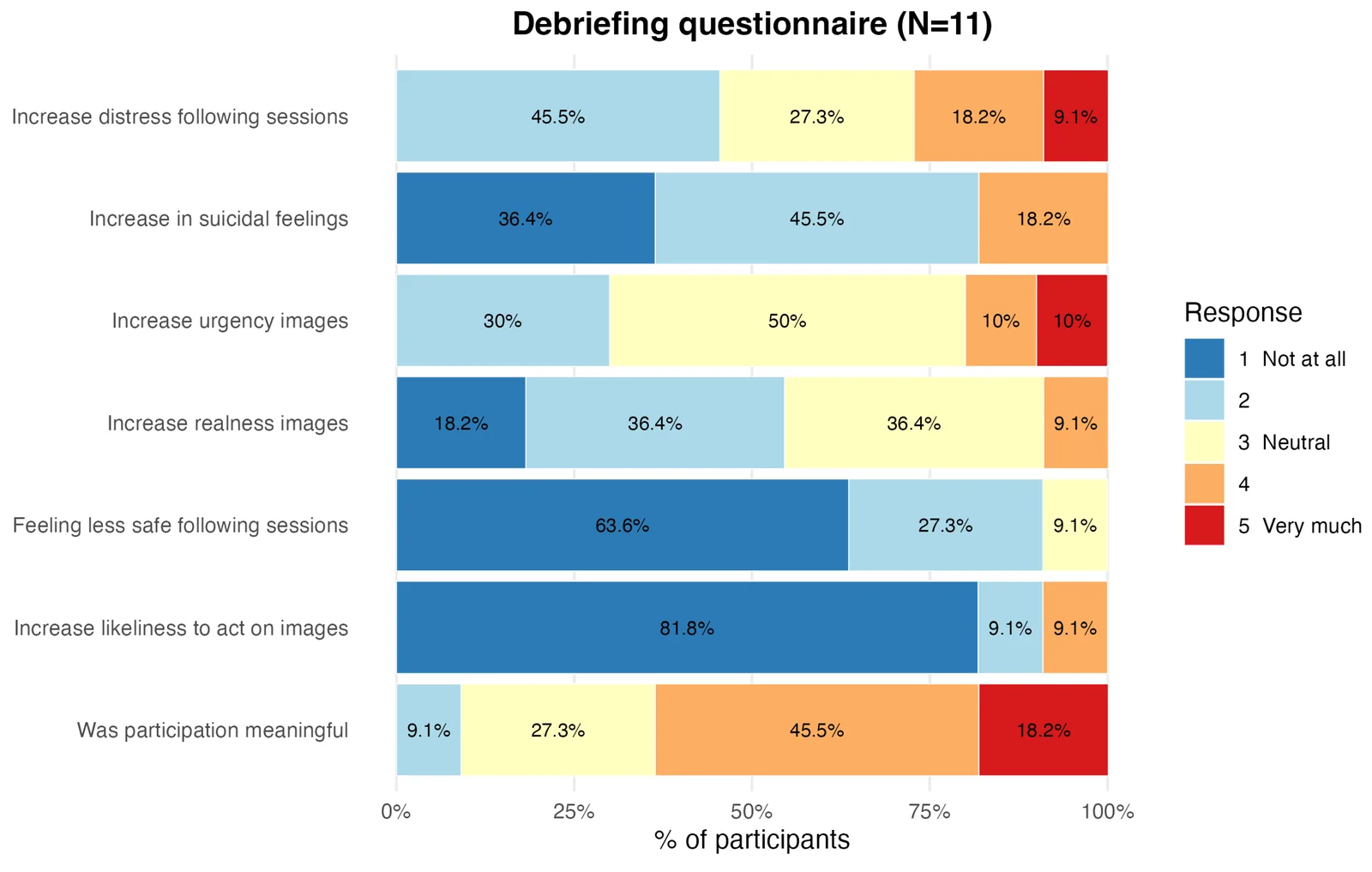
Participant-reported responses to debriefing questionnaire items assessing acceptability and safety of the imagery rescripting intervention (N = 11). Items were rated on a 5-point scale (1 = Not at all, 5 = Very much). For items 1–6 (increased distress, suicidal feelings, urgency, realness, feeling less safe, and likelihood to act), lower scores indicate better safety/acceptability outcomes; for the final item (meaningfulness of participation), higher scores indicate a more positive outcome. There was one missing score on increase in urgency of the suicidal mental images; thus, the denominator for that item was 10 instead of 11.

Based on the debriefing questionnaire, 8 of 11 participants (73%) who completed the imagery rescripting sessions indicated that the sessions were not distressing (score of 1, 2 or 3 on the “experiencing increase in distress following rescripting sessions” question), with 2 participants indicating the sessions were somewhat distressing (score of 4) and 1 participant finding the sessions very distressing (score of 5). Nine of 11 (82%) indicated that the sessions did not increase their suicidal feelings (score of 1 or 2), with 2 participants responding that the sessions somewhat increased their suicidal feelings (score of 4). Ten of 11 participants reported that the sessions did not increase their likelihood to act on their suicidal mental image. One participant did say that the session did somewhat increase this likelihood. For this participant, suicidal ideation severity decreased from baseline to follow-up (C-SSRS ideation severity score reduced from 5 to 1), within-session distress remained stable across two of their three intervention sessions and increased by one point (below the pre-specified ≥3-point threshold) in the third, and no clinician-initiated safety follow-up was recorded for this participant. None of the participants felt less safe after the imagery rescripting sessions (Figure 3). Thus, although three participants reported some degree of distress during the sessions (which can be expected when doing emotionally activating work such as ImRs), none reported feeling less safe afterwards, suggesting that transient emotional activation was generally tolerable and did not translate into perceived loss of safety. Both the induced distress and increase in feelings of unsafety criteria met the pre-specified >70% acceptability threshold.

#### 3.3.3. Safety

The study recorded two serious adverse events across the 11 participants. These related to preparatory acts towards engaging in self-harm and one suicide attempt. Both adverse events (AEs) met the seriousness criterion of ‘Other important medical event (not immediately life-threatening)’. The two SAEs occurred at different time points in relation to the intervention. The first occurred 31 days after the participant’s last ImRs session and was disclosed to the research assistant (RA) at the follow-up assessment. The second occurred between ImRs sessions, four days after the first ImRs session and before the second session, and was also disclosed to the RA at the follow-up assessment. Neither SAE occurred during an ImRs session.

In accordance with Sponsor procedures, events were reported to the Sponsor within 24 h and to the governing HREC through annual reporting. Severity, causality and expectedness were assessed through review of the events and medical records by the Principal Investigator, study clinician and Coordinating Investigator, with input from the treating team and senior ImRs supervisor. Consensus was that both events were consistent with participants’ clinical history and not temporally or mechanistically attributable to ImRs.

There was no new onset of self-harm behaviour (3 participants continued pre-existing self-harm behaviour in the period between baseline and follow-up, 3 discontinued). Rates of suicide attempts and new-onset self-harm behaviours in the current sample were lower than those observed in clients from the same youth mental health service from which participants were recruited (suicide attempts during episode of care: 11% (2/19); new-onset non-suicidal self-harm: 5% (1/19)), based on a subset of data from a file audit matched on duration of care (O’Gorman et al., 2023; Rice et al., 2022). None of the participants showed an increase in suicidal ideation from baseline to follow-up assessment, meeting the pre-specified threshold of ≤5%. Across all imagery rescripting sessions delivered, one participant showed a within-session increase in distress of 3 or more points from start to finish of one of their intervention sessions (9%), meeting the pre-specified threshold of <20%.

### 3.4. Exploratory Outcomes

The analyses examining effects of the intervention on suicidal ideation and other clinical variables are exploratory and should not be interpreted as evidence of efficacy, as this was an uncontrolled pilot with a small sample.

**Figure 4:**
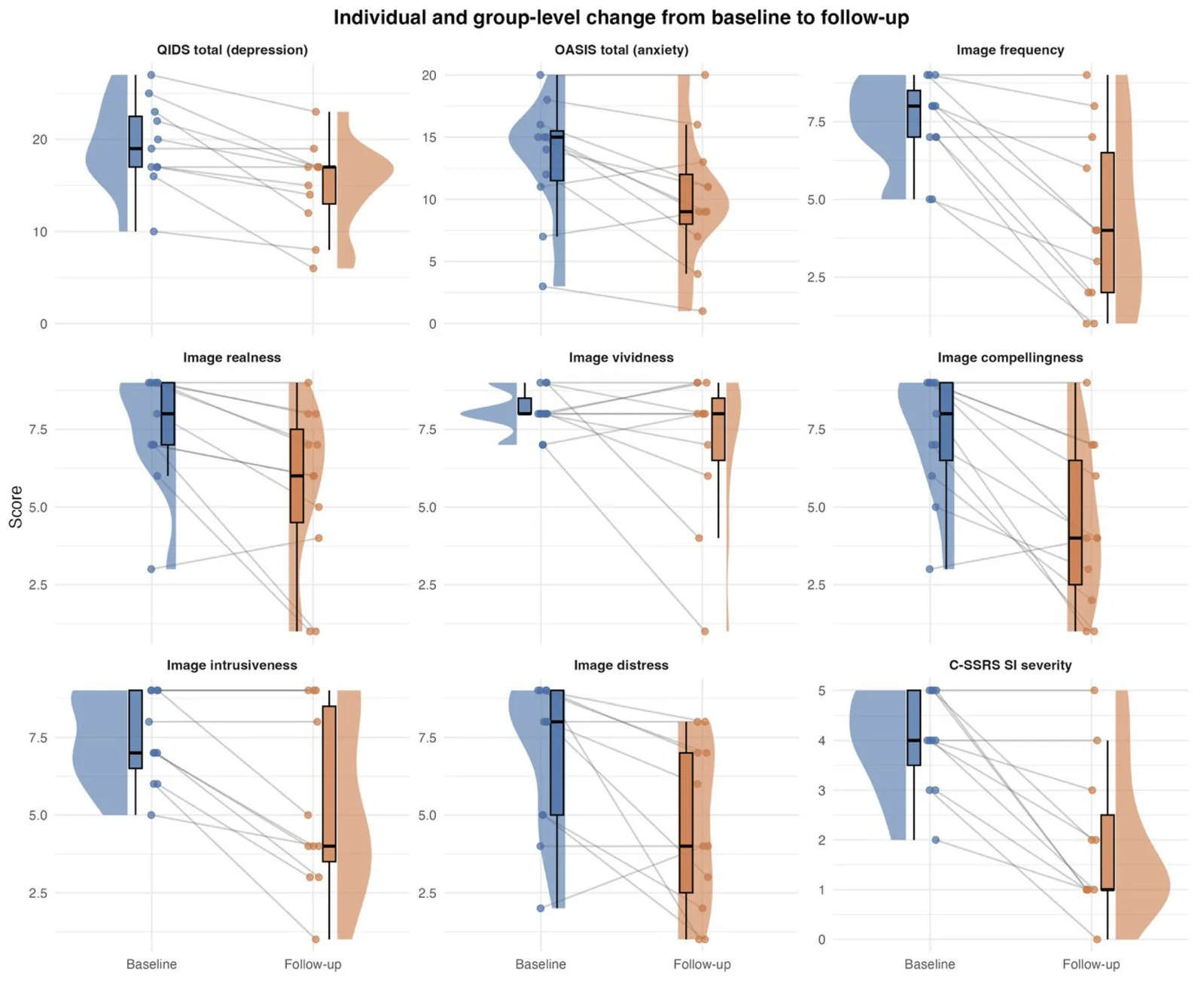
Individual and mean change in depressive symptoms, anxiety symptoms, suicidal ideation severity, and suicidal mental image characteristics from baseline to follow-up (post-intervention).

The suicidal mental images were reported as less frequent, less real and less compelling by 82% of participants; less distressing by 73%, less intrusive by 64%, and less vivid by 46% of participants (Cohen’s d’s ranging from −0.46 to −1.40) after the imagery rescripting intervention compared to baseline assessment, while none or very few participants reported a worsening in these characteristics (Table 3, Figure 4). Nine of 11 (82%) participants also showed a decrease in suicidal ideation severity, while two reported no change and none showed a worsening in suicidal ideation. Scores on suicidal ideation severity reduced on average by 62.5% (CI 37.5–87.5%), corresponding to a median decrease of 2.5 points (95% CI 1.5–3.5) on the C-SSRS suicidal ideation severity 0–5 scale. Severity of depressive symptoms (QIDS) decreased by 22.5% (CI 9.4–35.6%) and severity of anxiety symptoms by 24.7% (CI 6.3–43.1%). See Table 3 for confidence intervals, Cohen’s *d*’s and p values and Figure 3 displaying changes in each individual participant.

## 4. Discussion

This pilot study evaluated the feasibility, acceptability and safety of a brief, therapist-delivered imagery rescripting (ImRs) intervention that directly targeted suicidal mental imagery in help-seeking young people with mood disorder and suicidal mental imagery. Although the recruitment target was not met, intervention completion among eligible participants was high: 11 of 12 participants eligible for the intervention (92%) completed the introductory session, at least one rescripting session, and both research assessments. We note that whether the feasibility threshold was met depends on the denominator used. When using the consented denominator prespecified in Table 1 (N = 15), the completion rate was 73.3% and the >80% threshold was not met. By contrast, completion among those deemed eligible for the intervention after appropriate screening of the inclusion criteria of having an action-facilitating image was 91.7%. The discrepancy reflects two consented participants being excluded at baseline because their suicidal images were protective rather than action-facilitating and one participant who had been incorrectly consented despite not meeting the inclusion criterion of current suicidal ideation at screening, and were also found to be intoxicated during the intervention. This highlights the importance of clearly operationalising eligibility criteria and screening procedures in future trials, and suggests that feasibility in terms of retention once eligible is promising but that recruitment/eligibility dynamics should be considered when planning sample sizes. The prespecified acceptability and safety criteria were met. Most participants reported that rescripting was not distressing and did not increase suicidal feelings; no participant reported feeling less safe after the sessions, none showed an increase in suicidal ideation from baseline to follow-up, and only one showed a clinically meaningful within-session increase in distress. Importantly, this was not a low-risk analogue sample: participants were drawn from a specialist youth mood service and had substantial histories of suicidal and self-harm behaviours. Two serious adverse events occurred but were judged unrelated to the intervention. Failure to meet the recruitment target appeared to reflect study-delivery constraints and the narrow clinical eligibility window, including turnover among trained clinicians, competing case-management demands, and the requirement for recent action-facilitating suicidal imagery in the absence of acute suicide risk requiring more intensive clinical management, rather than poor uptake or retention once participants entered treatment. Future trials may therefore benefit from broader recruitment pathways and clinicians with protected responsibility for ImRs delivery, separate from case-management duties.

The study was not designed or powered to establish efficacy, but the exploratory outcomes provide a promising signal that warrants future evaluation in a fully powered randomised controlled trial. From baseline to follow-up, suicidal images became less frequent by 42.7%, less compelling by 40.7%, less intrusive by 28.0%, less real by 25.3%, and less distressing by 32.9%. Vividness showed a smaller, non-significant reduction. Suicidal ideation severity showed a large decrease of 62.5%, with smaller but still substantial reductions in depressive and anxiety symptoms. These changes are consistent with the proposed mechanisms of ImRs. Rather than simply suppressing an image, rescripting aims to alter its meaning by identifying the emotional or interpersonal needs represented within it and constructing an alternative sequence in which safety, adaptive coping and help-seeking can occur. For suicidal mental imagery, this process may also increase cognitive flexibility and problem-solving, reduce escape and avoidance-based responding, strengthen self-efficacy for alternative coping, and create more adaptive future-oriented imagery (Paulik et al., 2024).

These findings extend a small but growing literature showing that mental imagery is a viable intervention target for suicidality. In adults with depressive symptoms and distressing suicidal intrusions, Van Bentum et al. (2024) found that a brief cognitive dual-task intervention (mostly following an EMDR procedure) added to treatment as usual produced greater reductions in suicidal-intrusion severity (*d* = 0.60) and frequency, as well as suicidal ideation (*d* = 0.42), than treatment as usual alone. In university students with recent suicidal ideation or behaviour, Palmier-Claus et al. (2025) found the six-session Broad-Minded Affective Coping intervention, which uses guided imagery of positive autobiographical memories, to be feasible, acceptable and safe, with estimates for suicidal ideation favouring the intervention but were small and imprecise due to the limited sample size of this feasibility trial. These studies establish an important precedent for imagery-focused suicide interventions, but neither changes the content and personal meaning of the suicidal image itself. The present study therefore adds preliminary evidence that directly rescripting an action-facilitating suicidal mental image is acceptable and safe for young people. This is also consistent with Schmied et al. (2024), whose seven-participant case series found that a two-session ImRs intervention targeting self-harm-related imagery in adolescents was feasible and accompanied by improvements in self-harm, emotion regulation, self-efficacy, depression and anxiety. Together, these findings support progression to a controlled trial of suicidal-image rescripting while underscoring that efficacy remains unestablished.

The nature and characteristics of suicidal imagery reported in this study were broadly consistent with previous work, while also highlighting why suicidal imagery should not be treated as a unitary risk marker. Participants rated their images as highly frequent, vivid, real, intrusive and distressing, and all reported imagery of a future suicide attempt. Suicidal images were significantly more frequent than verbal suicidal thoughts and were numerically more intrusive. Similar vivid and future-oriented imagery has been described in young people with suicidality and self-harm. In our earlier study, 97% of help-seeking young people with current suicidal ideation reported suicidal imagery, most commonly involving a future suicide attempt or self-harm, and 44% reported that their imagery made them want to act (De Rozario et al., 2021). Susi et al. (2024) likewise found self-harm imagery in 98% of a clinical youth sample, commonly involving both past and future acts, with 88% reporting that their most significant imagery increased the likelihood of self-harm. However, the presence of suicidal imagery alone should not be assumed to indicate increased behavioural risk (Crane et al., 2012) and subsequent qualitative work shows that suicidal images vary in their affective and functional properties, including distress, comfort and escape-related meaning. Carey et al. (2026) similarly described a taxonomy spanning distressing and unwanted images as well as imagery experienced as comforting or problem-solving. Nilsson et al. (2026) identified both “suicide-approaching” imagery, such as intrusive looming images and flash-forwards that clarified a suicidal solution, and “suicide-preventive” imagery, including death-alienating and life-affirming images. This distinction was directly evident in our study: two otherwise eligible participants were excluded because their suicidal image functioned protectively rather than motivating action. Thus, selection of images for ImRs should be based on their individual meaning and role, not simply their suicidal content. Assessment should establish not only what is imagined, but whether the image increases motivation towards suicide, provides comfort or relief, or instead serves a protective or life-affirming function. This distinction is important because altering imagery that serves a protective function could be counterproductive. Careful, collaborative exploration of suicidal imagery is also consistent with qualitative findings that talking directly about suicide can be challenging but validating when supported by a strong therapeutic relationship (Duxbury et al., 2024).

The study had several limitations. First, the sample was very small, producing uncertain estimates and making the exploratory clinical outcomes vulnerable to sampling variability. Second, there was no control condition. Improvements may therefore reflect natural changes in suicidal crises, concurrent treatment, repeated assessment, therapeutic contact, or non-specific therapeutic effects. We did not systematically record detailed information on concurrent clinical care during the baseline–follow-up window (for example, medication changes, variation in treatment intensity, or crisis interventions), and therefore we cannot disentangle effects that are specifically attributable to ImRs from effects of contemporaneous treatment or non-specific therapeutic contact. Although most participants received only two ImRs sessions within a short timeframe, which makes large, therapy-driven changes in other treatment components less likely, we acknowledge that unmeasured changes in usual care could have contributed to the observed pre–post improvements. Nonetheless, the safety findings are encouraging given the high-risk clinical population. Previous file audits of the same specialist youth mood clinic found that 54.8% of young people had a lifetime history of suicide attempt at clinic entry (Rice et al., 2022), while 15.3% attempted suicide, 28.2% engaged in any self-harm and 67.9% experienced continued suicidal ideation during the episode of care (O’Gorman et al., 2023). These historical data provide useful clinical context, although the present sample is too small and methodologically different for direct comparison of event rates. Third, recruitment was constrained by clinician turnover and the relatively narrow eligibility window requiring recent action-facilitating suicidal imagery while excluding those requiring acute suicide-risk management. Fourth, treatment fidelity was not independently assessed, and future trials should incorporate formal monitoring of protocol adherence and competence to deliver ImRs. Finally, follow-up was short and longer-term safety and effectiveness still need to be established.

### Implications and Recommendations for Future Trials

Despite these limitations, the pilot provides useful signal-finding information to guide a definitive trial. A future study should use an adequately powered randomised design with a control comparator, blinded outcome assessment, longer follow-up, protected delivery by trained therapists, prospectively record concurrent treatments, and measures of image meaning, unmet needs, coping, self-efficacy and positive future imagery to test how change occurs. Safety procedures should be formalised with systematic adverse event capture at each contact, independent adjudication of relatedness, and consideration of an independent data safety monitoring mechanism for larger trials.

## 5. Conclusions

In this single-arm pilot, brief imagery rescripting targeted at action-facilitating suicidal mental imagery was deliverable within routine care and acceptable to participants, with encouraging short-term safety signals in a clinically high-risk sample. Observed reductions in suicidal-image characteristics and suicidal ideation are promising but exploratory and do not establish efficacy. A definitive randomised controlled trial that addresses the limitations noted above is required to determine whether ImRs produces clinically meaningful and durable reductions in suicidal imagery and suicidal outcomes. If replicated, targeting the content, meaning and function of suicidal imagery could add a novel brief, mechanism-focused treatment option for young people, which is urgently needed.

## Figures and Tables

**Table 1:** Feasibility, acceptability and safety criteria.

Outcome	Criterion
Feasibility	Recruitment target of N = 25 is met AND > 80% of consented participants complete 2 intervention sessions (i.e., first introductory session and one imagery rescripting session) as well as the baseline and follow-up assessment ^1^
Acceptability	>70% of participants indicate that the imagery-rescripting sessions were not considered distressing AND did not make them feel less safe
Safety	≤5% of participants showing increase in suicidal ideation from baseline to follow-up assessment. <20% of participants showing increase (3 or more points) in distress from start to finish of each imagery rescripting session. Not exceeding frequency of self-harm or suicide attempt compared to rates derived from our previous file audit of clients from the same youth mental health service (O’Gorman et al., 2023; Rice et al., 2022).

^1^ Of 15 participants who provided informed consent, 2 were subsequently excluded based on the baseline SCFI because their reported suicidal image was judged to be protective rather than action-facilitating and one was excluded because they had been incorrectly consented despite not meeting the inclusion criterion of current suicidal ideation at screening, and were also found to be intoxicated during the intervention; the feasibility numerator (completers) is therefore reported both relative to all consented participants (N = 15) and relative to those eligible for the ImRs intervention (N = 12).

**Table 2:** Participant characteristics at baseline.

Characteristic	N = 11 ^1^
Age (years)	19.1 (2.8)
Female, n (%)	8 (73%)
LGBTQI+, n (%)	8 (73%)
SUIS total score (tendency to think in images)	38.1 (11.7)
Major depressive disorder diagnosis (MINI)	11 (100%)
Past manic symptoms (MINI)	3 (27%)
Severity depressive symptoms (QIDS-SR-16)	19.4 (4.8)
Severity anxiety symptoms (OASIS)	13.3 (4.9)
Severity manic symptoms (ASRM)	2.8 (2.3)
Suicidal ideation severity (C-SSRS)	4.0 (1.0)
Presence of past actual suicide attempt (C-SSRS)	7 (64%)
Presence of past suicide actual/interrupted/aborted attempt (C-SSRS)	11 (100%)
Presence of non-suicidal self-harm (C-SSRS) past month	6 (55%)

^1^ Mean (SD); n (%).

**Table 3:** Changes in depressive and anxiety symptoms, suicidal mental image characteristics, and severity of suicidal ideation from baseline to follow-up.

Measure	N	Baseline M (SD)	Follow-Up M (SD)	% Change (95% CI)	Effect Size	Improved, n (%)	No Change, n (%)	Worsened, n (%)	*p*
QIDS total (depression)	11	19.4 (4.8)	15.0 (4.9)	−22.5% (−35.6%, −9.4%)	*d* = −1.16	9 (82%)	2 (18%)	0 (0%)	0.003
OASIS total (anxiety)	11	13.3 (4.9)	10.0 (5.3)	−24.7% (−43.1%, −6.3%)	*d* = −0.90	8 (73%)	1 (9%)	2 (18%)	0.014
Image frequency	11	7.5 (1.4)	4.3 (2.8)	−42.7% (−63.2%, −22.2%)	*d* = −1.40	9 (82%)	2 (18%)	0 (0%)	<0.001
Image realness	11	7.5 (1.9)	5.6 (2.7)	−25.3% (−43.7%, −6.9%)	*d* = −0.92	9 (82%)	1 (9%)	1 (9%)	0.012
Image vividness	11	8.1 (0.7)	7.0 (2.5)	−13.5% (−33.3%, 6.3%)	*d* = −0.46	5 (46%)	3 (27%)	3 (27%)	0.160
Image compellingness	11	7.4 (2.0)	4.4 (2.6)	−40.7% (−62.7%, −18.8%)	*d* = −1.25	9 (82%)	1 (9%)	1 (9%)	0.002
Image intrusiveness	11	7.5 (1.4)	5.4 (2.9)	−28.0% (−45.4%, −10.7%)	*d* = −1.09	7 (64%)	4 (36%)	0 (0%)	0.005
Image distress	11	6.9 (2.5)	4.6 (2.7)	−32.9% (−59.4%, −6.4%)	*d* = −0.83	8 (73%)	2 (18%)	1 (9%)	0.020
C-SSRS SI severity ^1^	11	4.0 (1.0)	1.9 (1.5)	−62.5% (−87.5%, −37.5%) = 2.5 points (−3.5, −1.5)	r = −1.00	9 (82%)	2 (18%)	0 (0%)	0.009

QIDS-SR-16 total score range: 0–27, OASIS total score range: 0–20, C-SSRS suicidal ideation severity range: 0–5, image characteristics range 1–9. ^1^ Change in C-SSRS suicidal ideation severity (an ordinal 6-point scale) was assessed with a Wilcoxon signed-rank test (% change and 95% CI from the Hodges-Lehmann pseudomedian difference; effect size = matched-pairs rank-biserial correlation, r). All other measures were assessed with paired-samples *t*-tests (% change and 95% CI from the mean difference; effect size = Cohen’s dz).

## Data Availability

The data sets generated during and/or analysed during the current study are available from the corresponding author on reasonable request.

## Abbreviations

The following abbreviations are used in this manuscript:

ImRs	Imagery rescripting
PTSD	Post-traumatic stress disorder
EMDR	Eye Movement Desensitisation and Reprocessing Therapy
HREC	Human Research Ethics Committee
ANZCTR	Australian New Zealand Clinical Trials Registry
SUDS	Subjective Units of Distress Scale
SCFI	Suicidal Flashforwards and Cognitions Interview
C-SSRS	Columbia-Suicide Severity Rating Scale
QIDS	Quick Inventory of Depressive Symptomatology
ASRM	Altman Self-Rating Mania Scale
OASIS	Overall Anxiety Severity and Impairment Scale
SUIS	Spontaneous Use of Imagery Scale
